# Synthesis and Characterization of Antibacterial Chitosan Films with Ciprofloxacin in Acidic Conditions

**DOI:** 10.3390/ijms242015163

**Published:** 2023-10-13

**Authors:** Dominik Sikorski, Piotr Rosiak, Łukasz Janczewski, Marek J. Potrzebowski, Dorota Kregiel, Sławomir Kaźmierski, Damian Neubauer, Beata Kolesińska, Justyna Frączyk, Anna Adamczyk, Zbigniew Draczyński

**Affiliations:** 1 Institute of Textile Materials and Polymer Composites, Lodz University of Technology, Zeromskiego 116, 90-924 Lodz, Poland; dominik.sikorski@dokt.p.lodz.pl; 2 Institute of Organic Chemistry, Faculty of Chemistry, Lodz University of Technology, Zeromskiego 116, 90-924 Lodz, Poland; piotr.rosiak@dokt.p.lodz.pl (P.R.); lukasz.janczewski@p.lodz.pl (Ł.J.); justyna.fraczyk@p.lodz.pl (J.F.); 3 Centre of Molecular and Macromolecular Studies, Polish Academy of Sciences, Sienkiewicza 112, 90-363 Lodz, Poland; marek.potrzebowski@cbmm.lodz.pl (M.J.P.); kaslawek@cbmm.lodz.pl (S.K.); 4 Department of Environmental Biotechnology, Faculty of Biotechnology and Food Sciences, Lodz University of Technology, Wólczańska 171/173, 90-924 Lodz, Poland; dorota.kregiel@p.lodz.pl; 5 Department of Inorganic Chemistry, Faculty of Pharmacy, Medical University of Gdańsk, 80-210 Gdansk, Poland; damian.neubauer@gumed.edu.pl; 6 Faculty of Materials Science and Ceramics, AGH University of Science and Technology, A. Mickiewicza 30 Av., 30-059 Krakow, Poland; aadamcz@agh.edu.pl

**Keywords:** chitosan, ciprofloxacin, antibacterial activity, drug, structural investigation

## Abstract

This work presents the results of research on obtaining chitosan (CS) films containing on their surface ciprofloxacin (CIP). A unique structure was obtained that not only gives new properties to the films, but also changes the way of coverage and structure of the surface. The spectroscopic test showed that in the process of application of CIP on the surface of CS film, CIP was converted from its crystalline form to an amorphic one, hence improving its bioavailability. This improved its scope of microbiological effect. The research was carried out on the reduction of CIP concentration during the process of CIP adhesion to the surface of chitosan films. The antibacterial activity of the CS films with and without the drug was evaluated in relation to *Escherichia coli* and *Staphylococcus aureus*, as well as *Candida albicans* and *Penicillium expansum*. Changes in the morphology and roughness of membrane surfaces after the antibacterial molecule adhesion process were tested with atomic force microscopy (AFM) and scanning electron microscopy (SEM). Structural analysis of CS and its modifications were confirmed with Fourier-transform spectroscopy in the infrared by an attenuated total reflectance of IR radiation (FTIR-ATR) and solid-state nuclear magnetic resonance (NMR).

## 1. Introduction

Infections are diseases caused by various organisms [1]. Bacterial infections are the most commonly discussed and can cause mild lesions that usually go away on their own, but can also cause serious, life-threatening conditions such as sepsis [2].

Not taking care of even the smallest wound can cause serious illness. Chronic wound infections caused by various bacteria are a difficult medical challenge. Gram-negative bacteria with multidrug resistance attract undue attention as there are currently no commercially available dressings to combat them [3]. There is therefore an urgent need to develop new materials, such as films and dressings, capable of inactivating Gram-negative and Gram-positive bacteria. Natural polymers are increasingly used for this purpose. One of them is chitosan derived from chitin.

Chitin (Ct) and chitosan (CS) belong to the group of natural polysaccharides. They occur in the shells of marine invertebrates, as well as in the cell walls of insects and fungi. The main source for the industrial production of chitin is waste from the fishing industry. Both natural forms of Ct and CS, as well as those subjected to surface modification, are used in biotechnology, pharmacy, medicine, and other fields. These polymers have structures that can be modified quite easily, creating a wide spectrum of derivatives that increase their application in many fields [4,5]. Due to its non-toxicity, antibacterial activity, bio-adhesive characteristics, biodegradability, and excellent biocompatibility, CS is widely applied in the biomedical field, as a drug carrier and as an antimicrobial, antioxidant, antitumor, and wound-dressing agent [6,7,8,9,10,11,12,13]. CS has the ability to form a variety of morphological structures, which can be helpful to contribute to this aim, such as films, fibers, hydrogels, membranes, nanoparticles, and microbeads [14].

From a molecular point of view, CS is a cationic polysaccharide consisting of β (1 → 4)-2-amino-2-deoxy-D-glucopyranose and β (1 → 4)-2-acetomido-2-deoxy-D-glucopyranose or a homopolymer of β (1 → 4)-2-amino-2-deoxy-D-glucopyranose. Its chemical and physical properties depend mainly on its degree of deacetylation (DD), molecular weight (MW), and amino groups (-NH_2_) presence in a molecule [15]. The main parameters responsible for the number of amino groups in the chain are the molecular weight and the degree of deacetylation, including the same environmental parameters such as ionic strength, pH, and temperature [16,17].

CS has three different reactive functional groups: -NH_2_ at C-2, -OH at C-6, and -OH at C-3. They are often used to enhance the bioactivity of CS. Due to them, it is possible to combine CS with other compounds to broaden its range of usage [18]. This can be utilized to strengthen microbial immunity. The antimicrobial properties of CS can be improved by chemical modification of the CS. The usage of the two functional groups (-NH_2_) and (-OH) offers enormous possibilities for chemical modification. It is even viable to obtain several functional derivatives through reactions such as sulfonation, amination, and carboxymethylation. Quaternization increases the positive charge density of CS and may also improve its antimicrobial efficacy due to the enhancement of its polycationic character in the chitosan salt [19,20].

In addition to the antimicrobial activity of the polymer itself, as well as its modifications, it can be combined with various molecules, including antibacterial drugs. One such medicine is ciprofloxacin (CIP). Ciprofloxacin is a fluoroquinolone antibiotic that acts within a broad spectrum of Gram-positive and Gram-negative bacteria. CIP inhibits the activity of DNA gyrase and topoisomerase IV, which results in the degradation of chromosomal DNA and the lowering of gene expression [21]. It can be used as an additive compound in wound-dressing materials obtained by electrospinning [22].

CIP is also applied to obtain various structures. As stated by Egorov et al., 2022, the CIP molecule, due to the properties of its carboxyl functional group, can be conjugated to the primary amino group of chitosan. A conventional carbodiimide method is employed to form amide bonds by conjugating a carboxyl group and a primary amine group. This method uses the commonly available carbodiimide DCC (N,N-dicyclohexylcarbodiimide) [23]. In the works of Arauzo et al. and Cui et al., CIP was also attached to chitosan. Arauzo used CS sub-micron particles loaded with CIP, which were generated by electrospray, while Cui presented CIP-encapsulated nanofibrous membranes prepared by electrospinning with the use of core-shell structured fibers with CIP [24,25]. Pignatello et al. showed the generation of microparticles and used the injection method [21]. Others have used hydrogels as well as various composites for CIP incorporation [26,27,28,29,30].

The defined hypothesis of this research was the following: it is possible to generate membranes (in the form of films) and assign them antimicrobial properties with chemical modification. The aim of this research was to apply a chemotherapeutic from the group of second-generation quinolones (fluoroquinolones) with a bactericidal effect (as an active substance) to modify membranes made of CS in such a way that they displayed antimicrobial properties. In the first stage, membranes (films) were prepared. Native CS (with free amine group) did not display bacteriostatic properties. It was assumed that, in the following step, CS films were chemically modified with an active substance, which enabled the desired effect. In the final part, an additional process of conditioning the modification of CIP-CS materials by using acetic acid vapors was conducted in order to enhance the bacteriostatic effect by acquiring positively charged ammonium groups (CS salt).

It was expected that this modification would improve the membrane coverage with antibiotics and enlarge the scope of its intended effect. The active substance, which is a crystalline drug, attaches itself to the surface, taking an amorphic form, giving the added advantage of lowering the energy needed to dissolve it. It was expected that the drug would be permanently bound to the membrane through a weak bond between chitosan and ciprofloxacin. Additionally, it was assumed that the chitosan membrane coverage with an antibiotic would be strengthened via treatment with acetic acid vapors. The use of acetic acid causes ciprofloxacin to dissolve and increase surface coverage by spilling onto the film, which increases the area of action of ciprofloxacin. The use of organic acid, as can be seen from microbiological studies, significantly increases the bacteriostatic effect for certain strains in relation to samples where ciprofloxacin was not used, as well as the control sample.

## 2. Results

### 2.1. FTIR-ATR Analysis

**Figure 1:**
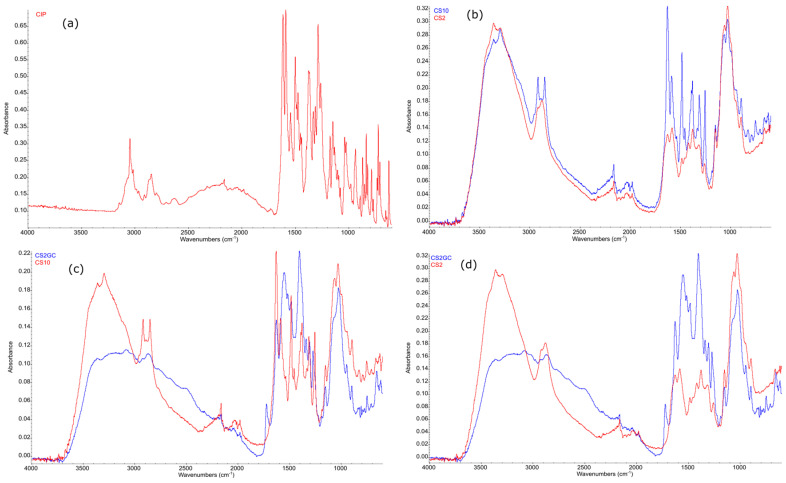
FTIR spectra of chitosan films modified with CS-CIP physical adducts and additionally treated with acetic acid vapors: (**a**) CIP; (**b**) superimposed spectra of unmodified CS (CS2) and CS-CIP physical adducts (CS10); (**c**) superimposed spectra of CS10 and CS10 additionally treated with acetic acid vapors; (**d**) superimposed spectra of CS (unmodified) and CS10 additionally treated with acetic acid vapors (CS2GC).

In the first stage of testing, attempts were made to verify if chitosan film modifications with CIP (physical adducts) and additional treatment with acetic acid vapors resulted in a positive outcome. The prepared materials were put to the by using the FTIR-ATR method (Figure 1). Sample spectra of unmodified chitosan (CS), CIP-modified chitosan (CS-CIP physical adducts), and CS-CIP physical adducts additionally treated with acetic acid vapors are presented below.

Comparing the FTIR-ATR spectra of unmodified CS films and CS-CIP physical adducts in the form of a film, changes in the intensity of the characteristic CS signals can be observed, which implies that the CIP was fixated on the surface by intermolecular interactions. A shift of signals in the range of 1700–1200 cm^−1^ was found, and this is consistent with the literature data for ciprofloxacin-loaded chitosan nanoparticles [31,32].

Changes in the FTIR-ATR spectra of unmodified CS (CS2) and CS-CIP physical adducts (CS10) additionally treated with acetic acid vapors are visible in the 1800–1700 cm^−1^ range, which indicates the formation of small amounts of chitosan acetate on the surface. Additionally, changes in signal intensity in the 1707–1040 cm^−1^ range (Pandey et al., 2012; Sikorski et al., 2022) are visible, which illustrates that CIP and residues of acetic acid are present on the CS film layers [33,34].

When summarizing the FTIR-ATR spectra of CS film incubated with CIP solution (CS10) with CS film formed by treatment with CIP and acetic acid vapors (CS2GC), the widening of signals characteristic to CS can be observed, which might be the result of CIP, CS acetate, and CS signals overlapping. The intensity of CS acetate signals in the FTIR-ATR spectrum is dominant within this spectrum, as shown in Figure 1c, which is probably the result of the reaction occurring between free amino groups of CS with acetic acid vapors.

### 2.2. Solid-State NMR Studies

Solid-state NMR studies were conducted with ^13^C CP/MAS (cross-polarization magic angle spinning) analysis of individual components, which were used for the preparation of CS-CIP physical adducts in the form of a film.

**Figure 2:**
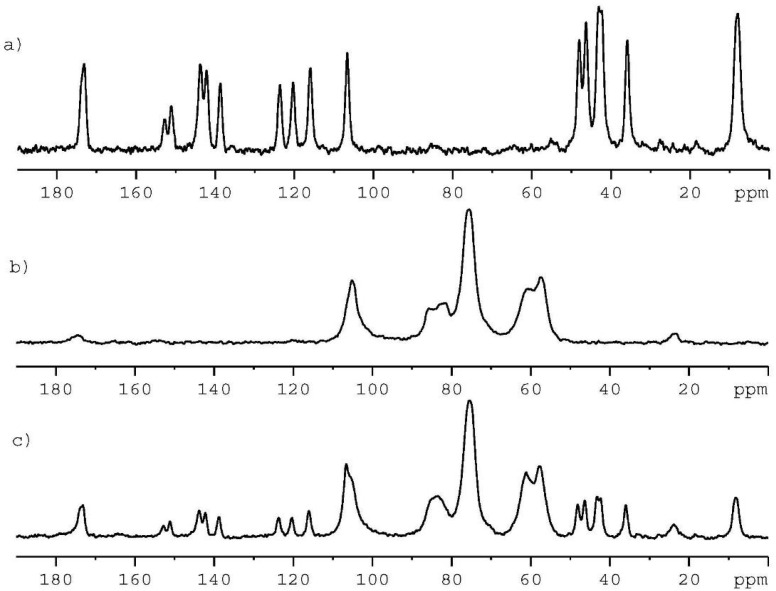
Shown are the 100 MHz ^13^C CP/MAS NMR spectra for (**a**) pure crystalline CIP, (**b**) CS, and (**c**) physical mixture of CIP and CS with a molar ratio 1:1. All spectra were recorded with a spinning rate of 8 kHz at ambient temperature.

Figure 2a shows the 100 MHz ^13^C CP/MAS spectrum of CIP recorded at a spinning rate of 8 kHz. From previous studies, it is known that CIP occurs in various crystallographic forms: as anhydrous crystals and as two hydrates with different water content. These forms were studied in detail by Mafra and colleagues [35].

^13^C NMR resonances for each form were assigned using experimental 2D NMR techniques and theoretical GIPAW calculations. Based on the literature data, we can conclude that the sample used in our project is an anhydrous form with Z’ equal to one (Ref. Code: UHITOV).

Figure 2b displays the ^13^C CP/MAS spectrum of CS. Studies describing the ^13^C NMR spectra of chitosan in the solid state are very extensive, and many articles have been published to date. The CS used in our project shows a typical pattern, with clearly visible signals of the carbohydrate unit (50 ppm–115 ppm region) and weak signals representing the acyl group (carboxylate at δ = 176 ppm and methyl carboxylate at δ = 23 pm).

The ^13^C CP/MAS spectrum of the physical mixture of CIP and CS with a molar ratio of 1:1 obtained by manual grinding of two components is shown in Figure 2c. As one can see, this spectrum is the simple sum of the spectra shown in Figure 2a,b. The overlapping of the carboxylate components of CIP and CS at δ = 176 ppm is worth noting.

In the next step, we confronted our ^13^C CP/MAS NMR results with ^19^F MAS NMR data. From the point of view of NMR spectroscopy, fluorine-19 is a “friendly”, easy-to-detect nucleus. ^19^F has a nuclear spin I of 1⁄2 with a high gyromagnetic ratio and contains 100% naturally occurring fluorine. The ^19^F nucleus is the third most receptive nucleus of NMR, after the ^3^H and ^1^H nuclei.

Fluorine-19 is very sensitive to the chemical environment, and depending on the molecular structure, the ^19^F signal can be detected in the range of 800 ppm. In an asymmetric environment, it is characterized by high chemical shift anisotropy (CSA). In CSA, the liquid phase is eliminated by fast overall tumbling, and only the isotropic signal is detected. In the solid state, CSA’s effect is reduced or eliminated by fast magic angle sample spinning. In most cases, in order to reduce the number of spinning sidebands, the sample should be recorded with a spinning rate over 20 kHz [36,37,38].

**Figure 3:**
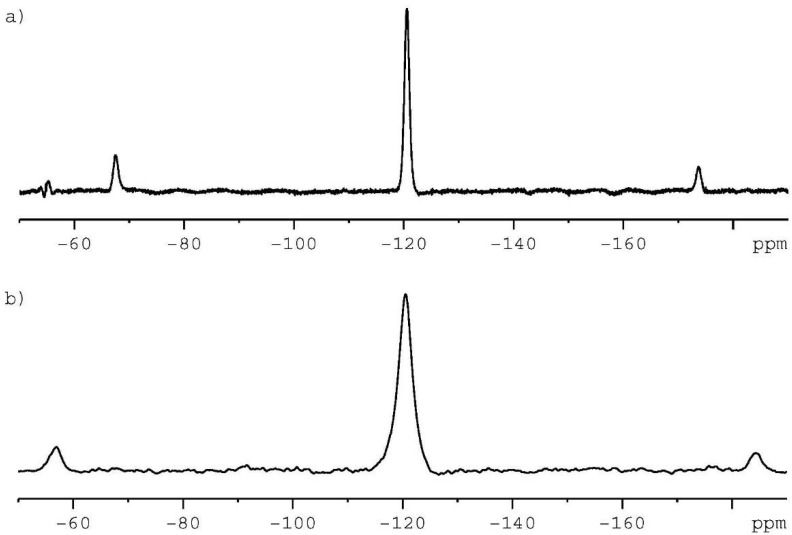
Shown is the 376.55 MHz ^19^F MAS NMR spectra of (**a**) pure, crystalline CIP, and (**b**) physical mixture of CIP and CS with molar ratio 1:1. Spectrum (**a**) was recorded with a spinning rate of 20 kHz at ambient temperature. Spectrum (**b**) was recorded with spinning rate 24 kHz at ambient temperature. In both cases, eight scans were obtained.

Figure 3a shows the ^19^F MAS spectrum of pure crystalline CIP (see Figure 2a) recorded with a spinning rate of 20 kHz. Only eight scans were sufficient to acquire good-quality data. The isotropic signal found at δ = −120 ppm is symmetrically surrounded by spinning satellites (signals at δ = −72 ppm and δ = −168 ppm). The spectrum confirms that one molecule of CIP is an asymmetric unit, as concluded from X-ray tests for anhydrous form. Figure 3b displays the ^19^F signal for CIP mixed with CS (see Figure 2c). The spectrum was recorded at a spinning rate of 24 kHz, hence the spinning satellites are seen in a different position than in Figure 3a. The isotropic signal, as in the previous case, occurs at δ = −120 ppm. It should be emphasized that in both cases the widths of the ^19^F resonance lines are significantly different. For pure CIP the full width at half height (FWHH) is equal to 610 Hz, while for the CIP/CS mixture, the FWHH is found to be 1150 Hz. This difference suggests that during the grinding of components partial amorphization of CIP can occur. In addition, it is noteworthy that such broadening of signals was not observed on the ^13^C CP/MAS spectra. This means that the 19F nucleus is a very sensitive probe (more sensitive than the ^13^C nucleus) not only to internal effects, but also to distant interactions.

**Figure 4:**
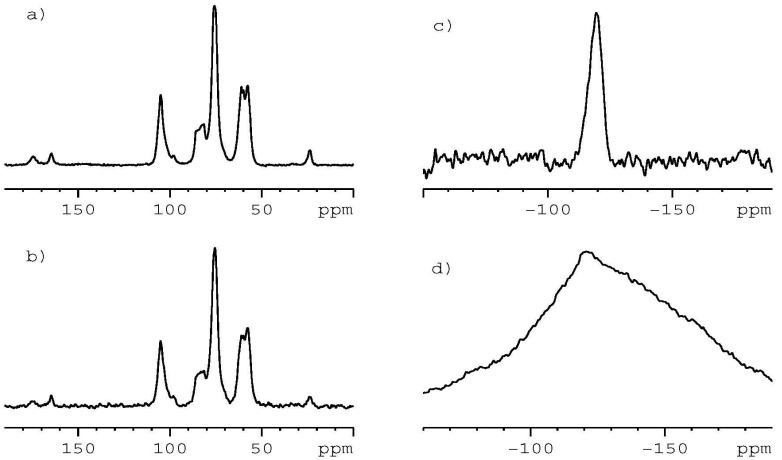
(**a**) ^13^C CP/MAS NMR spectrum of CS 100 impregnated with CIP recorded with spinning rate 8 kHz. (**b**) ^13^C CP/MAS NMR spectrum of CS 30 impregnated with CIP recorded with spinning rate 8 kHz. (**c**) ^19^F MAS NMR spectrum of CS 100 impregnated with CIP recorded with spinning rate 20 kHz. (**d**) ^19^F MAS NMR spectrum of CS 30 impregnated with CIP recorded with spinning rate 20 kHz.

In the final step, we investigated the samples of the chitosan films impregnated with CIP. We employed two kinds of chitosan correspondingly labeled CS 100 and CS 30. Figure 4a,b shows the ^13^C CP/MAS spectra for CS 100 impregnated with CIP and CS 30 impregnated with CIP, respectively.

From the analysis of the ^13^C NMR spectra, it is clear that resonances representing CIP are not observed. The only new signal occurred at δ = 166 ppm, which is not representative of CIP. This result can be interpreted as follows: (a) CIP is not incorporated into CS during impregnation; (b) CIP is incorporated in a very small amount into CS, but the ^13^C NMR probe is not sensitive enough to detect NMR signals; and (c) CIP is incorporated in a very small amount into CS but is very amorphous. For such cases, the resonance lines are very broad and difficult (or impossible) to measure. In order to explain these doubts in further studies, we employed a more sensitive NMR probe than the ^13^C-^19^F nucleus. Figure 4c shows the ^19^F MAS NMR spectrum for the sample CS 100 impregnated with CIP recorded with a spinning rate of 20 kHz. The data were acquired overnight (5 k scans). The signal of CIP is clearly seen. Its FWHH is equal to 2480 Hz, much more when compared to the crystalline sample of CIP or a mixture CIP/CS. The ^19^F MAS spectrum for sample CS 30 impregnated with CIP is even more spectacular (Figure 4d). In this case, FWHH is over 20 kHz (Figure 4d).

In summary, in line with the NMR results, we can say that CIP is included in CS and is strongly amorphous. Unfortunately, our measurements do not allow us to make quantitative conclusions and determine the amount of CIP in CS.

### 2.3. AFM Analysis

**Figure 5:**
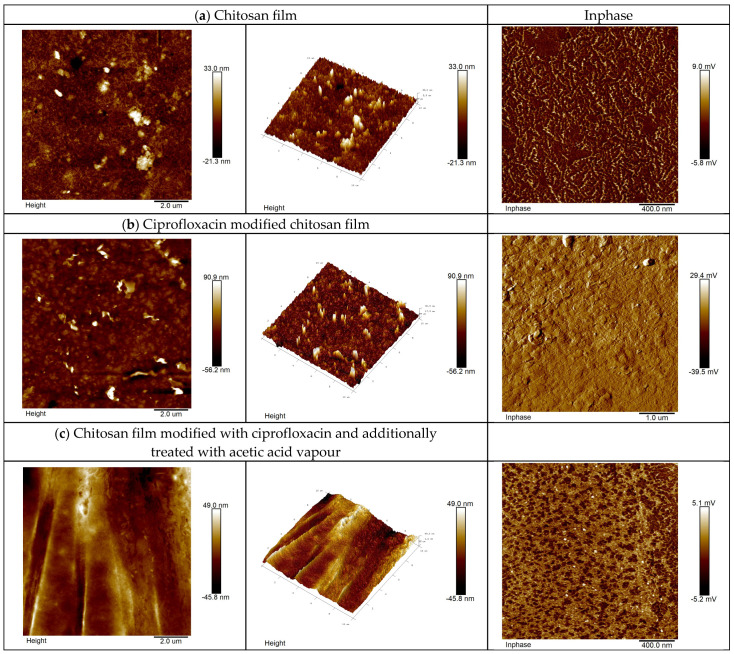
Changes in the structure of CS films surface after the application of CIP sodium salt and acetic acid vapors.

AFM image analysis (Figure 5) indicates that the applied modification of CS films with CIP (Figure 5b) and with acetic acid vapors treatment (Figure 5c) substantially affects the morphology of the surface.

The presence of the adduct of CIP on the CS film surface can be observed (Figure 5b). The additional treatment of CS film modified with CIP (physical adduct) and acetic acid vapors results in complete coverage of the film surface (Figure 5c). AFM images illustrate the topography in narrow layers, as shown in Figure 5a–c. The topography can be characterized with different parameters to quantify the mean squared (RMS) of the surface roughness [39]. The RMS value of the surface roughness is calculated from the cross-sectional profile or from the surface area. RMS surface roughness obtained for the narrow chitosan layers was 33 nm, 90.9 nm, and 49 nm, respectively. The RMS surface roughness of CS film increases as a result of coating it with CIP, which can be ascribed to the combination of two materials: CS and CIP. The surface roughness implies a smoothing mechanism by surface diffusion. The activity of the acetic acid causes the spread of CIP on the surface of the sample.

Additionally, the test in phase mode was conducted. In phase is related to mechanical–viscosity and probe–surface interactions, which are in turn related to the chemical composition of the surface. Every alteration in the chemical composition affects the change of signal, which allows for “separating” the phases, and their distribution on the sample surface. This is especially well visible for samples of chitosan film modified with CIP (physical adduct) and with acetic acid vapors (Figure 5c). This effect is slightly less visible for the samples of CS films modified with CIP (physical adduct) (Figure 5b). This highlights the areas with various properties (different colors on scans); they also have a disparate shape to the one for the sample (Figure 5c). The AFM technique proved to be very helpful in assessing the surface properties of the materials studied, as also noted by other authors researching chitosan films. According to the literature data, Almalik et al. [40] applied the AFM method to test CS nanoparticles, which were covered with hyaluronic acid (HA). In this test, AFM was used to determine the presence of HA on CNPs. The thickness of HA coverage on CPS was estimated to be around 20–30 nm in a dry state. Fauzi et al. [41] used AFM to test the morphology of nanocomposite surfaces of thin chitosan/maghemite films in order to potentially apply the films to optical detection of Hg^2+^ ions by surface plasmon resonance. Rubina et al. [42] used atomic force microscopy as a base tool for morphology tests of CS films. The tested CS films were taken out from acetic acid solutions of different concentrations as well as CS films containing gold and silver.

### 2.4. SEM Image Analysis

**Figure 6:**
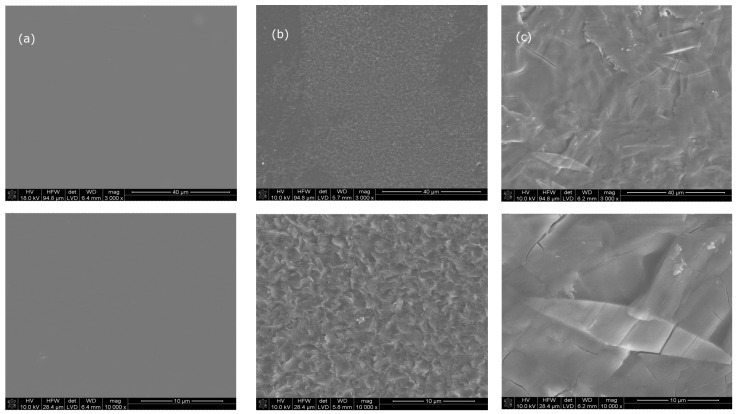
Microstructure images of CS film (**a**) treated with CIP sodium salt (**b**) and subsequently treated with acetic acid vapors (**c**) in different magnifications 3000× and 10,000×.

Also in the SEM images (Figure 6), clear changes on the surfaces of the samples modified with CIP alone, as well as with additional modification with acetic acid vapors (Figure 6b,c), can be seen, in comparison to unmodified chitosan film (Figure 6a).

CS-CIP physical adducts and additionally treated with acetic acid vapors: (a) CIP, (b) superimposed spectra of unmodified CS (CS2) and CS-CIP physical adduct (CS10), and (c) superimposed spectra of CS10 and CS10 additionally treated with acetic acid vapors.

SEM image analysis confirms the presence of CIP on the film surface of CS-CIP physical adducts (Figure 6b) and CS-CIP physical adducts and additionally treated with acetic acid vapors (Figure 6c). The surface of the unmodified CS film is smooth and flat, while the modified samples show a rough surface with characteristic features, the observation is consistent with the data from the AFM analysis. Rathore et al. also demonstrated a similar relationship [43]: with the increase in CIP concentration, the porosity of the film also increased. For the samples additionally treated with acetic acid vapors, the changes in CIP solid structure can be noted showing a more uniform distribution of the drug on the surface of the film. These changes result in better coverage of the CS discs with CIP in relation to films not treated with acetic acid vapors. This is probably related to the dissolution of CIP in an acidic environment during the incubation of the sample in acetic acid vapors as well as the occurrence of the anticipated extra effect of CS swelling in an acidic environment. There are also a lot of SEM analyses concerning the similar observation [44] in which chitosan with clay for composites formation was tested or the work of Cazón et al. [45] where they obtained chitosan-tea tree essential oil (TTEO) films as a new biodegradable material for a potential future application in food packaging.

### 2.5. Analysis of CIP Adhesion on Surface of Chitosan Discs 

Measurements of changes in CIP concentration due to CIP adhesion on the surface of chitosan discs were carried out using the RP-HPLC method.

**Figure 7:**
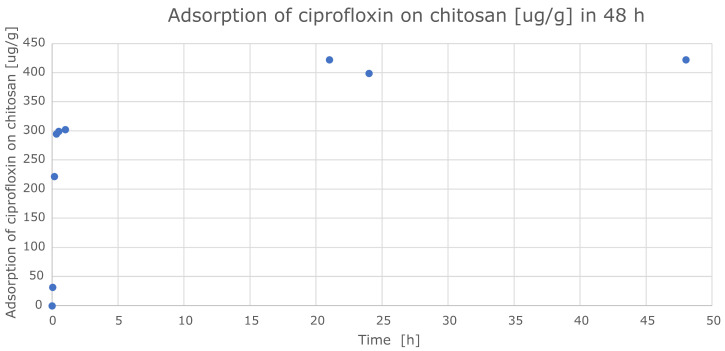
Dependence of CIP solution depletion (C_0_ = 15 × 10^−4^ M) during adhesion to chitosan disc surface (CS2, m = 0.15 g) as a function of time (the test was performed at 25 °C).

Figure 7 shows the dependence of the CIP loss from the solution during the adhesion of the antibacterial drug on the surface of the chitosan disc and the duration of the process.

In the graph showing the depletion of CIP, an intensive sorption process can be observed within 12 h, while a balance of sorption and desorption processes on the surface of the CS disc is observed after 21 h. For the tested CS film with a thickness of 50 mm, the amount of adsorbed CIP is determined at the level of 420 mg CIP per 1 g of chitosan.

CIP adsorption on chitosan was performed for many different variants of chitosan composites. In all cases, CIP adsorption changed the structure of chitosan materials [46,47,48,49,50]. Usually, modified chitosan materials are used as materials for the removal of water pollution, including drug residue.

### 2.6. Analysis of Antibacterial and Antifungal Activity of CIP-Chitosan Adduct in Film Form

The antimicrobial activity resulting from the measurement of microbial growth inhibition zone φ (mm) is demonstrated in Table 1.

The molecular weight of CS and the degree of deacetylation significantly influence its biological activities [51]. CS was described as a strong antimicrobial against Gram-positive, Gram-negative bacteria, and fungi [52]. However, the antimicrobial properties of CS are dependent on relevant factors, including type of microbial strain, pH level, and structural properties [53].

Introducing new moieties to the polymer chain can improve the biological properties of CS. The available literature describes different CS modifications by catechol [54], quaternary ammonium groups [55], or Schiff bases [56]. Various synthetic approaches are available for obtaining CS derivatives, such as direct modification, enzymatic reactions, or chemical grafting [57]. It should be noted that executing a successful multistep organic synthesis of complex molecules can be a challenging process. The main concern may be that certain functional groups can interfere with the reaction, resulting in undesired products. This situation can be avoided by using protective groups that temporarily mask the functional groups that would interfere [58].

The tested CS materials showed diversified antimicrobial activity. None of them were active against tested fungal strains. CS with different molecular weight and viscosity showed no significant antimicrobial activity, except for the CS6 modification with weak activity against Gram-negative bacteria *E. coli*. However, the chemical modifications of chitosan created interesting antimicrobial effects. Very interesting results were obtained for the modification of CS with CIP—the efficient antibacterial effect was noted in relation to both Gram-negative and Gram-positive bacteria. This fact is worth noting because CIP is known as an antibiotic with good activity against most Gram-negative bacteria, but its action against Gram-positive bacterial strains may be moderate or weak [59].

Modifications of chitosan with acetic acid, in turn, resulted in a weaker antimicrobial effect, even though acetic acid is known to have a broad antibacterial activity. This substance has been commonly used in medicine for more than 6000 years for the disinfection of wounds and especially as an antiseptic agent in the treatment and prophylaxis of the plague. The microbiological spectrum of acetic acid is wide, even when at a low concentration of 3% [60,61].

**Figure 8:**
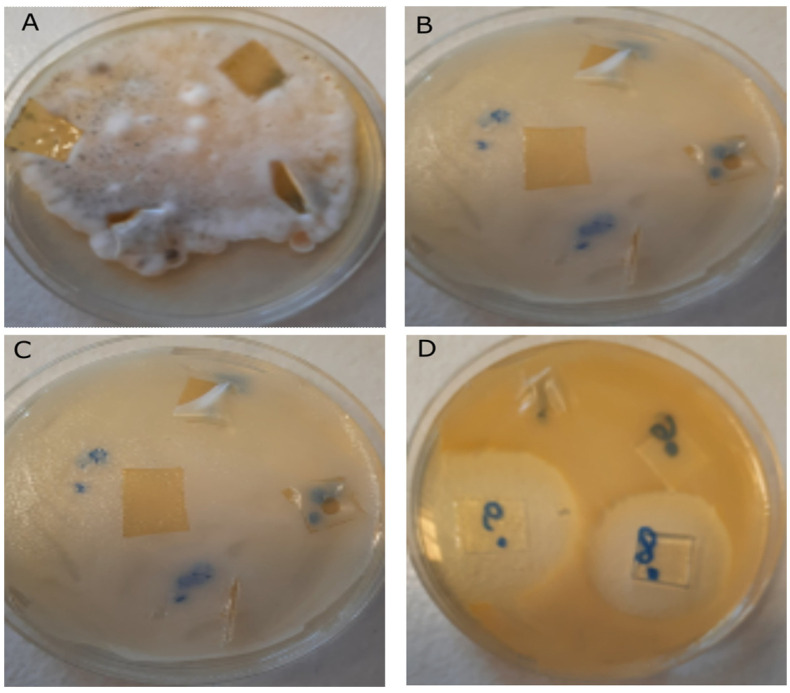
Biocidal effect for the individual bacterial strains and fungi: (**A**) *Penicillium expansum* CS6, CS1C–CS3C; (**B**) *Candida albicans* CS6, CS1C–CS3C, (**C**) *Escherichia coli* CS6, CS1C–CS3C; (**D**) *Staphylococcus aureus* CS6, CS1C–CS3C; inhibition zone clearly visible.

Better results can be obtained by modifying CS with acetic acid and an additional active substance as can be seen in Table 1 and Figure 8. Lately, chitosan derivatives with acetic acid and guanidinium groups were synthesized and characterized by ^13^C NMR, FTIR spectroscopies, thermogravimetry, and elemental analysis. Both N-guanidinium CS acetate and N-guanidinium CS chloride displayed high antimicrobial activity in comparison with neat CS [62]. In addition, freeze-dried CS acetate with incorporated silver nanoparticles effectively controlled the growth of bacteria [63]. These data suggest that synergistic combinations of chitosan acetate and other antimicrobials lead to improved antibacterial efficacy of CS.

This effect was also confirmed in our research. The best antibacterial effect was achieved by modifications of CS with CIP and acetic acid. These modifications lead to strong synergistic action against both Gram-negative and Gram-positive bacteria.

The findings suggest that the prepared antibacterial material with drug delivery properties could be an appropriate candidate for many medical and hygienic applications.

## 3. Materials and Methods

### 3.1. Preparation of Chitosan Solutions—Substrates for Obtaining Chitosan Films

Substrates/reagents were measured and added in the following order: 190 mL of distilled water, 6 g of acetic acid (99.5–99.9% PURE P.A. POOCH Polish Chemicals Reagents, Gliwice, Poland), 4 g of CS. The following chitosan samples were used: CS1 from Pol-Aura, Poland, molecular weight 890 kDa; CS2 from Pol-Aura, Zawroty, Poland, molecular weight 30 kDa; CS3 from Qingdao Chibo Biotech, Qingdao, China, molecular weight 150 kDa; CS4 from Qingdao Chibo Biotech, China, molecular weight 325 kDa, CS5 from Qingdao Chibo Biotech, China, molecular weight 84 kDa; CS6 from Qingdao Chibo Biotech, China, molecular weight 60 kDa. Subsequently, the suspension was mixed with a mechanical stirrer (200 rpm) for 24 h until a homogeneous solution was achieved.

### 3.2. GPC/SEC Analysis—Determination of Molecular Weight of Chitosan

Molecular studies of chitosan, including the determination of molar mass distribution function (MMD), the average values of molar masses (Mn, Mw), and the polydispersity (Mw/Mn), were performed using the gel permeation chromatography/size exclusion chromatography (GPC/SEC) method in accordance with the described procedure [64].

### 3.3. Preparation of Chitosan Films

A solution of previously prepared chitosan (3.5 g) was weighed and poured onto the Petri dish (50 × 12 mm). The Petri dishes were put in a laboratory dryer, and they were dried until the complete solidification of the polymer at the temperature of 70 °C. The dry film was detached from the Petri dish with 2% solution (NaOH PURE P.A. POOCH Polish Chemicals Reagents, Gliwice, Poland). The films were then rinsed in distilled water at 60 °C. After that, wet chitosan films were put on PET foil and covered with filter paper.

### 3.4. Modification of Chitosan Films by Acetic Acid Vapor Treatment

The process of acetic acid vapor treatment of chitosan films was carried out in a desiccator and was based on the condensation of acetic acid vapors on the surface of the chitosan films. Acetic acid from Avantor Performance Materials Poland POOCH Polish Chemicals Reagents was used for the tests. The procedure described in the literature was used [33].

### 3.5. Modification of Chitosan Films with CIP—Procedure of Coating Discs (Preparation of Physical Adducts of Chitosan and CIP)

The modification of chitosan films with CIP for the purpose of testing the antibacterial activity.

The chitosan films before the coating with CIP sodium salt were treated with 5 × 10^−2^ M NaOH solution for 2 h in order to relax the structure of the polymer. After this time, the discs were washed with ultrapure water and treated with CIP sodium salt solution. CIP (AC58172, Biosynth Ltd., London, UK) sodium salt solution (10 mL of 5 × 10^−4^ M) was applied to the films. Ultrapure water was used as a solvent. The films were treated with CIP salt solution for 12 h. In order to remove the excess CIP, membranes were washed with 3 × 10 mL ultrapure water, and then they were dried in the vacuum desiccator until constant mass was achieved.

### 3.6. Quantitative Assessment of CIP Content in Physical Chitosan-CIP Adduct by Using RP-HPLC Method

The amount of adsorbed CIP was designated by the measurement of CIP loss from 2 mg/100 mL solution in 48 h, in which a chitosan disk (film) with a diameter of 6 cm was immersed. The tests were performed by the HPLC method. For each measuring point, 6 analyses were performed. These results were the basis for obtaining the average value for a given measurement point. The analyses were carried out after 3, 6, 10–60 min and after 21, 24, and 48 h. CIP absorption was measured at the 278 nm wavelength. Analytical method: 10–100%, 10 min, 0.5 mL/min, 25 °C, mobile phase (A) water and (B) acetonitrile, both solvents containing 0.1% (*v*/*v*) formic acid. Column Agilent, Zorbax SB-C18, 4.6 × 50 mm, 1.8 μm. The volume of the applied sample was 20 μL. RP-HPLC, Waters e2695 Separations Module, detector Waters 2998 PDA, software Empower 3.

### 3.7. Analysis of Surface of Physical Adduct of CS-CIP by AFM Method

The measurements were taken in PeakForce Tapping mode, under the AFM Multimode 8 microscope made by Bruker, Bruker Nano Surfaces Division, Santa Barbara, CA USA. AFM scans were obtained by registering 512 lines for each image and by scanning with a frequency of around 1 kHz.

The same AFM analysis parameters were used for the samples of CS films treated with CIP and acetic acid vapors.

### 3.8. Analysis of Surface of Physical Adduct of CS-CIP by SEM Method

Samples were attached to microscopic stages with conductive carbon tape. Subsequently, a layer of 10 nm carbon was used to cover the samples. A Leica EM ACE 600 (Leica Microsystems GmbH, Wetzlar, Germany) sputtering coater was used for this purpose. Microstructure observations and chemical analysis were conducted with the use of Nova NanoSem 200 (FEI, Eindhoven, The Netherlands) high-resolution scanning electron microscope equipped with EDS energy dispersive spectrometer (EDAX, Tilburg, The Netherlands). Observations and analyses were carried out in low-vacuum conditions (60 Pa), with an LVD detector in secondary electron mode and with an accelerating voltage of 10 kV.

### 3.9. Analysis of Physical Adduct of CS-CIP and CS-CIP Films Treated with Acetic Acid Vapors by FTIR Method

The molecular structures of the chitosan nonwovens were analyzed using a Nicolet 6700 FTIR spectrometer (Thermo Scientific, Waltham, MA, USA) in attenuated total reflectance (ATR) mode, with a diamond crystal (incidence angle 45°). The measurement conditions were as follows: the resolution was 2 cm^−1^, the range of infrared (IR) radiation was 4000–600 cm^−1^, and 32 scanning procedures of each sample were carried out using IR radiation to obtain the absorption spectra. The absorption spectra of the samples were plotted in the system A = f(1/1), as a basis for the interpretation of the molecular nonwoven structure.

### 3.10. Analysis of Physical Adduct of CS-CIP by NMR Method

^13^C NMR experiments were registered on a Bruker Avance III 600 spectrometer, with an operational frequency of 150.93 and 600.15 MHz for ^13^C and ^1^H, respectively. In each experiment, a sample was placed in a 4 mm ZrO_2_ rotor and spun with 8 kHz spinning speed. ^13^C CP/MAS spectra were measured with a repetition time of 10 s at ambient temperature. A sample of U-^13^C, ^15^N-labeled histidine hydrochloride was used to set the Hartmann–Hahn condition for ^13^C, with a proton 90° pulse length of 4 μs. For cross-polarization, the nutation frequency was 50.5 kHz for ^13^C, with a ^1^H ramp shape from 90% to 100% and a ^1^H nutation frequency of 62.5 kHz. In all cases, the SPINAL-64 decoupling sequence with a ^1^H pulse length of 3.6 μs was applied. The ^13^C chemical shift was referenced indirectly by using adamantane (resonances at 38.48 and 29.46 ppm) as an external secondary reference.

^19^F MAS NMR spectra were recorded on a 400-MHz Bruker Avance III spectrometer, equipped with a ^1^H, ^19^F, broadband-resonance 2.5 mm MAS probe head operating at 376.55 MHz for fluorine. The 90° pulse duration was set to 2.25 μs. For the experiment with ^19^F decoupling, a π–pulse scheme was used with a 180° pulse equal to 6 μs. The collected spectra were externally referenced to CFCl3 (0 ppm). All spectra were recorded and processed with a Bruker TopSpin 3.1 program.

For the analysis of ^13^C CP MAS and ^19^F MAS RO NRM in solid phase (mixture of solid CS and solid CIP), two samples containing 10 mg and 50 mg of CIP were prepared, and both samples were filled up to 0.3 g with CS. To homogenize the samples, the solids were thoroughly mixed and pounded in a mortar.

To perform the ^13^C CP MAS NMR analysis of the physical adduct of CS-CIP, CS film samples in the form of discs were incubated in 10 mL of 15.1 × 10^−4^ M CIP for 48 h.

### 3.11. Assessment of Antimicrobial Activity of Physical Adduct of CS-CIP and CS-CIP Films Treated with Acetic Acid Vapors

Antimicrobial properties of the chitosan samples against bacteria: Gram-positive *Staphylococcus aureus* ATCC 6538 and Gram-negative *Escherichia coli* ATCC 8739 as well as fungi: *Candida albicans* ATCC 10231 and *Penicillium expansum* LOCK 0535 were tested.

The agar diffusion method was performed in accordance with methods for antimicrobial preparation, inoculum preparation, inoculation, incubation, and data interpretation [65].

All experiments were carried out in triplicate. Microbial suspensions were prepared by suspending 3–5 well-separated colonies from tryptic soy agar (TSA) (bacteria) or malt extract agar (MEA) (fungi) plates in 3 mL of Ringer solution, and the turbidity was adjusted to the 0.5 McFarland standard (Densitometer D1, Merck, Darmstadt, Germany).

The suspensions were used directly as the final seed in the diffusion method. For diffusion tests, agar plates were inoculated with the suspensions of the microorganisms described above by uniform swabbing in three directions to form a lawn. Then sterile 5 mm × 5 mm chitosan squares (sterilized under UV light 265 nm, 1 h per each side) were placed on the surface of the inoculated agar plates with sterile forceps. In a similar manner, control samples, positive control—a paper square soaked with ciprofloxacin solution and negative control—a paper square without antibiotics, were placed on the agar plates. The plates were incubated at 37 °C for 24 h (bacteria) and 25 °C for 3 days (fungi), and the diameters of the zones of growth inhibition were measured.

For the antibacterial activity tests, a set of chitosan films, physical adduct of CS-CIP, and CS-CIP films treated with acetic acid vapors, in the form of films were used (Table 2).

### 3.12. Additional Analysis of Determination of Degree of Deacetylation of Chitosan 1H-NMR Spectroscopy—Figures and Table Are in Supplementary Materials Section

The chemical structures and DDA (degree of deacetylation) of the initial chitosan, as well as of the chitosan after acid treatment, were determined by 1H-NMR spectroscopy Table S1 and Figure S1 analysis using a Bruker AM 400 (Bruker Corporation, Billerica, MA, USA) spectrometer, in a mixed solvent containing C_2_DF_3_O_2_ (Trifluoroacetic acid-d) from Sigma-Aldrich (St. Louis, MO, USA) and D_2_O (deuterium oxide). The samples were prepared as follows: 10 mg of the chitosan salts was dispersed in D_2_O and, after homogenization, was dissolved by adding C_2_DF_3_O_2_ to finally obtain 50 μL C_2_DF_3_O_2_ in 9.950 mL D_2_O. When the solution formed, measurements were made at 323 K using 32 scan pulse accumulations. The degree of chitosan deacetylation was calculated using the following formula, where DS is the degree of substitution as in [66] formula number 12.

## 4. Conclusions

In conclusion, we successfully produced chitosan (CS) films containing ciprofloxacin (CIP) on their surface. Spectroscopic studies showed that in the process of applying CIP to the surface of CS films, CIP was converted from a crystalline to an amorphous form, which improved its bioavailability. This improved the extent of its microbial activity. A study was conducted to measure changes in CIP concentration during the adhesion process of CIP on the surface of chitosan films. The balance of sorption and desorption processes on the surface of the CS disc was determined after 21 h. For the CS film tested with a sample thickness of 50 mm, the amount of CIP adsorbed was determined to be approximately 420 mg/g. The antimicrobial properties of the modified chitosan films were tested against Gram-positive bacteria, *Staphylococcus aureus* ATCC 6538; Gram-negative bacteria, *Escherichia coli* ATCC 8739; and fungi, *Candida albicans* ATCC 10231 and *Penicillium expansum* LOCK 0535. Antimicrobial activity against *Escherichia coli* and *Staphylococcus aureus* was demonstrated for CIP-CS-modified films. Changes in the morphology and surface roughness of the films after antibiotic adhesion were studied by atomic force microscopy (AFM) and scanning electron microscopy (SEM). The RMS surface roughness obtained for the narrow chitosan films was 33 nm, 90.9 nm, and 49 nm, respectively. By comparing the FTIR-ATR spectra of unmodified CS and CIP-modified chitosan films, changes in signal intensity characteristic for CS were observed, suggesting that the antibiotic was fixed on the surface by intermolecular forces.

## Figures and Tables

**Table 1:** Results of microbiological analysis of the CIP adduct on the surface of the chitosan discs.

Sample Symbol	*Escherichia coli* ATCC 8739	*Staphylococcus aureus* ATCC 6538
CS 1	0	0
CS 2	0	0
CS 3	0	0
CS 4	0	0
CS 5	0	0
CS 6	30	0
CS 1C	45	36
CS 2C	47	36
CS 3C	50	36
CS 4C	52	36
CS 1G	24	0
CS 2G	0	0
CS 3G	0	0
CS 4G	0	0
CS 5G	24	0
CS 6G	32	0
CS 1CG	58	45
CS 2CG	60	60
Control—ciprofloxacin 32 μg/mL	42	31

0: No zone of inhibition.

**Table 2:** Characteristics of chitosan used for the modifications and method of modifications. The time of vaporization was 120 min.

Lp	Sample Name	Mn, g/mol	Mw g/mol	Mw/Mn	Description
1	CS 1	94,700	209,500	2.21	without modifications
2	CS 2	13,600	28,900	2.12	without modifications
3	CS 3	5380	154,200	2.87	without modifications
4	CS 4	85,700	216,200	2.52	without modifications
5	CS 5	113,100	189,800	1.68	without modifications
6	CS 6	53,900	129,200	2.40	without modifications
7	CS 1C	-	-	-	Ciprofloxacin modification
8	CS 2C	-	-	-	Ciprofloxacin modification
9	CS 3C	-	-	-	Ciprofloxacin modification
10	CS 4C	-	-	-	Ciprofloxacin modification
11	CS 1G	-	-	-	modification with acetic acid vapors
12	CS 2G	-	-	-	modification with acetic acid vapors
13	CS 3G	-	-	-	modification with acetic acid vapors
14	CS 4G	-	-	-	modification with acetic acid vapors
15	CS 5G	-	-	-	modification with acetic acid vapors
16	CS 6G	-	-	-	modification with acetic acid vapors
17	CS 1CG	-	-	-	Ciprofloxacin modification and subsequent modification with acetic acid vapors
18	CS 2CG	-	-	-	Ciprofloxacin modification and subsequent modification with acetic acid vapors

## Data Availability

Part of the data is stored at the Institute of Textile Materials and Polymer Composites.

## Supplementary Materials

The following supporting information can be downloaded at: https://www.mdpi.com/article/10.3390/ijms242015163/s1.
