# Supplementary material for: Synthesis and Characterization of Antibacterial Chitosan Films with Ciprofloxacin in Acidic Conditions
Source: Int J Mol Sci. 2023 Oct 13;24(20):15163. doi: 10.3390/ijms242015163 (PMC10606985; doi:10.3390/ijms242015163)
Supplement: Supplementary file 1 [file ijms-24-15163-s001.zip › ijms-2610008-supplementary.pdf]

Figure S1. Analysis of chitosan's based on 1H- NMR Spectroscopy

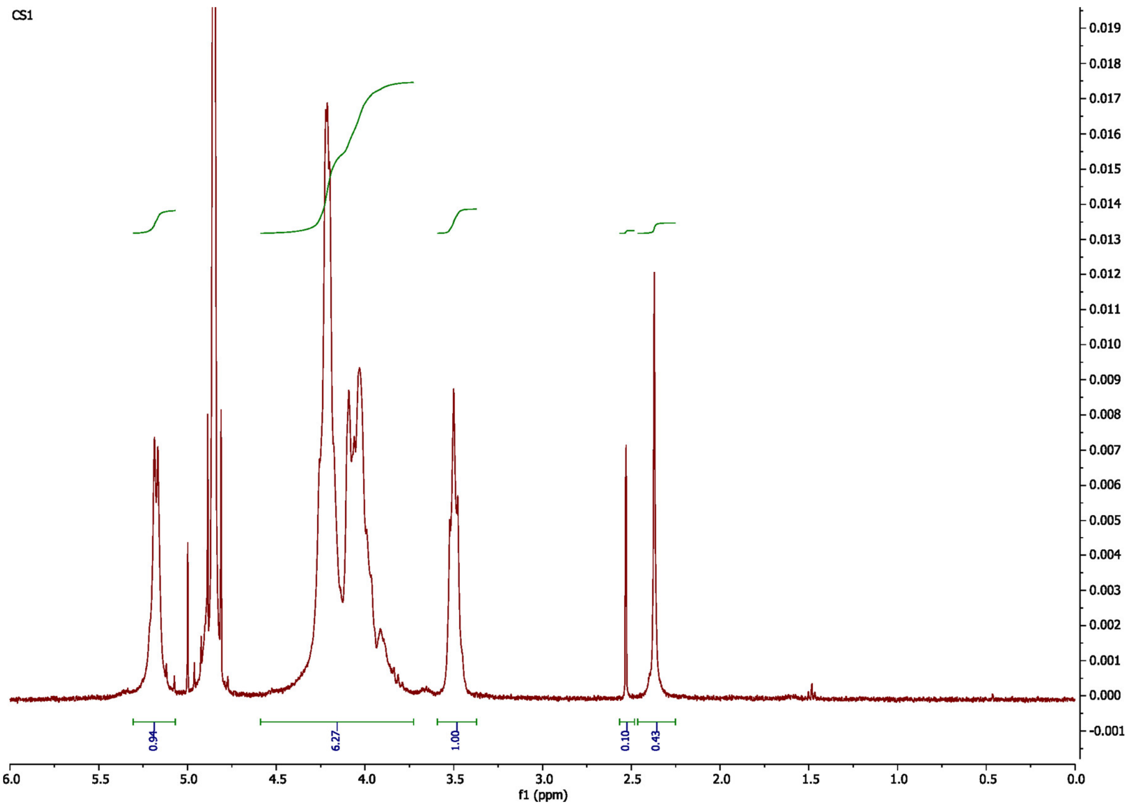

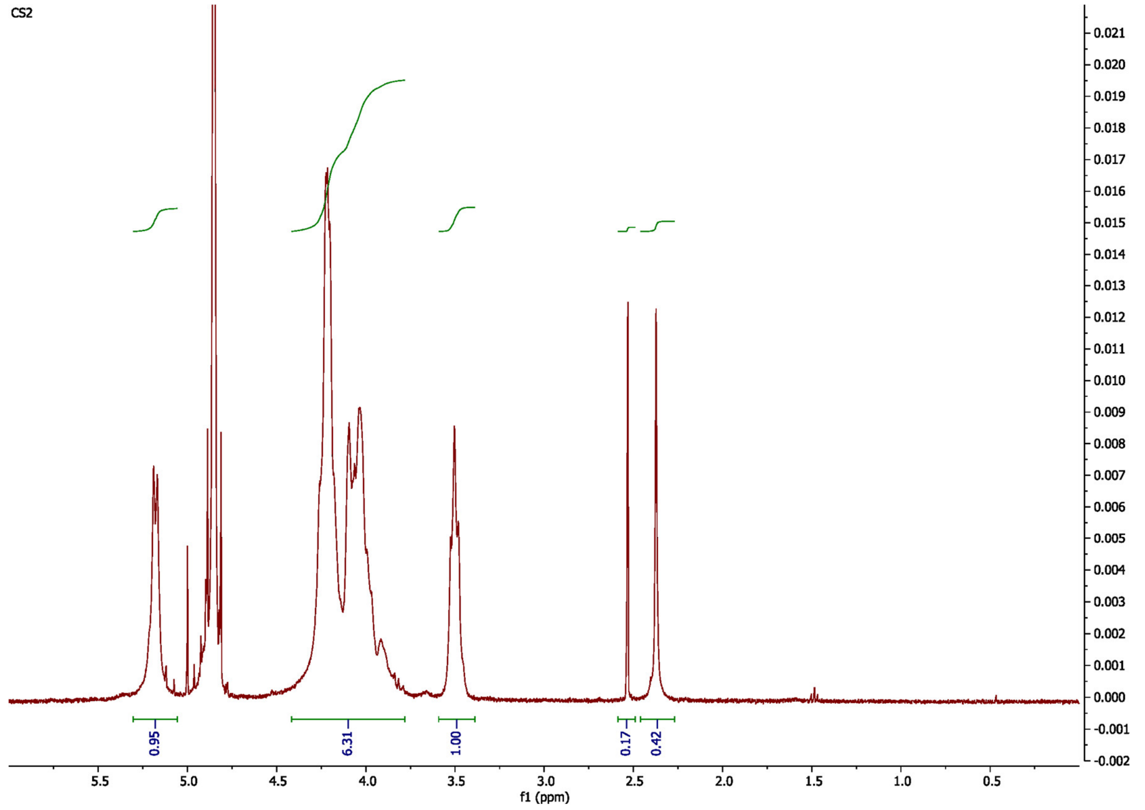
CS2
0.95
6.31
1.00
0.17
0.42
5.5 5.0 4.5 4.0 3.5 3.0 2.5 2.0 1.5 1.0 0.5
f1 (ppm)
0.021 0.020 0.019 0.018 0.017 0.016 0.015 0.014 0.013 0.012 0.011 0.010 0.009 0.008 0.007 0.006 0.005 0.004 0.003 0.002 0.001 0.000 -0.001 -0.002

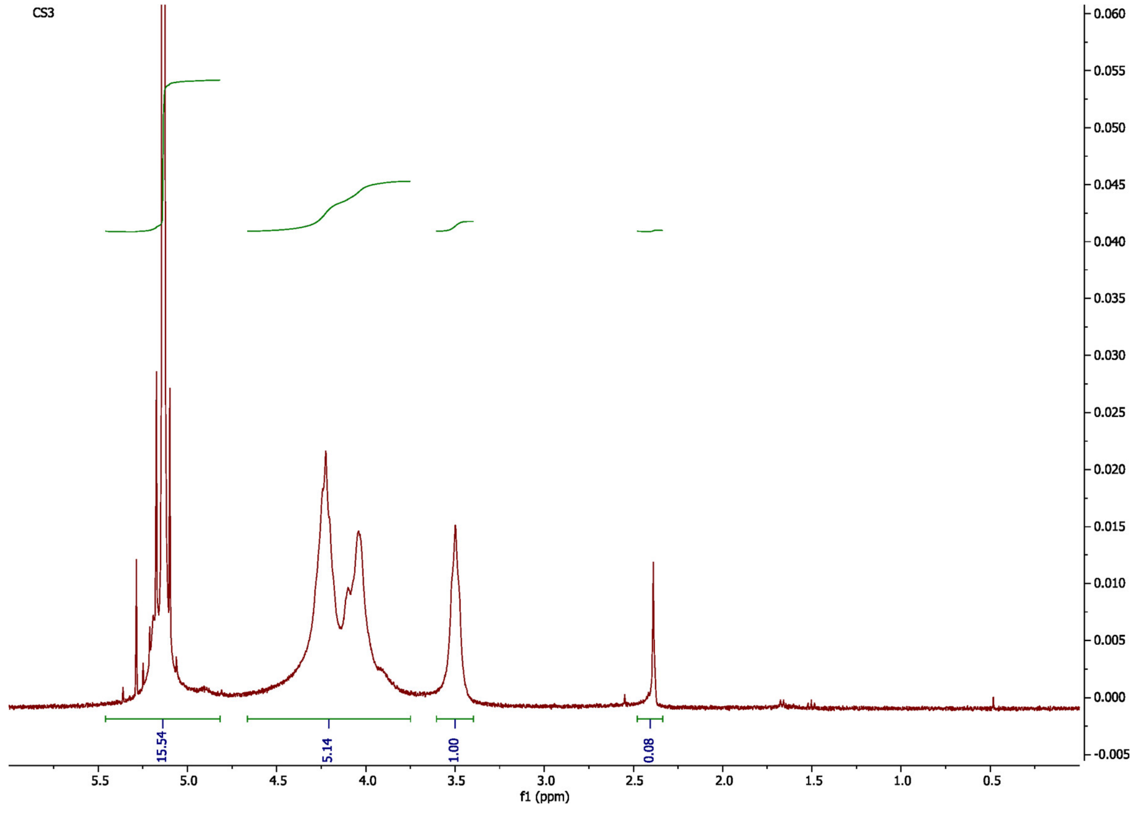
CS3
15.54
5.14
1.00
0.08
5.5 5.0 4.5 4.0 3.5 3.0 2.5 2.0 1.5 1.0 0.5
f1 (ppm)
0.060 0.055 0.050 0.045 0.040 0.035 0.030 0.025 0.020 0.015 0.010 0.005 0.000 -0.005

CS4

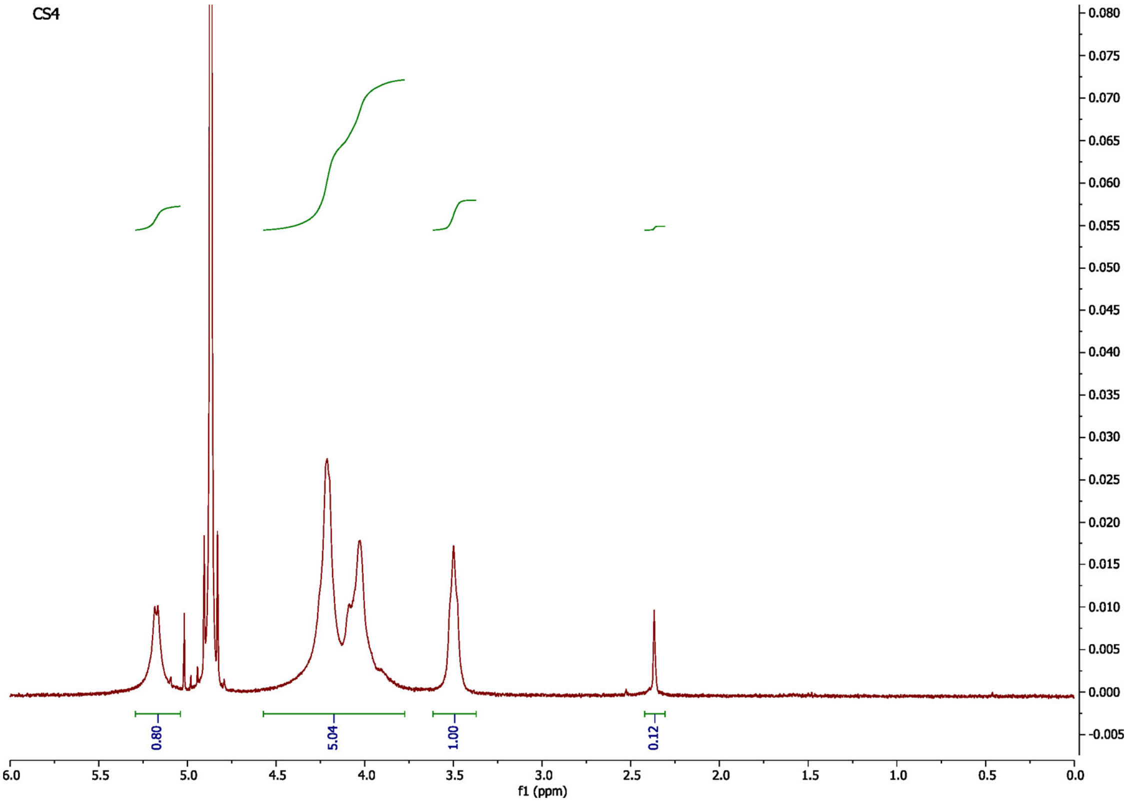
0.80
5.04
1.00
0.12
f1 (ppm)

CS5

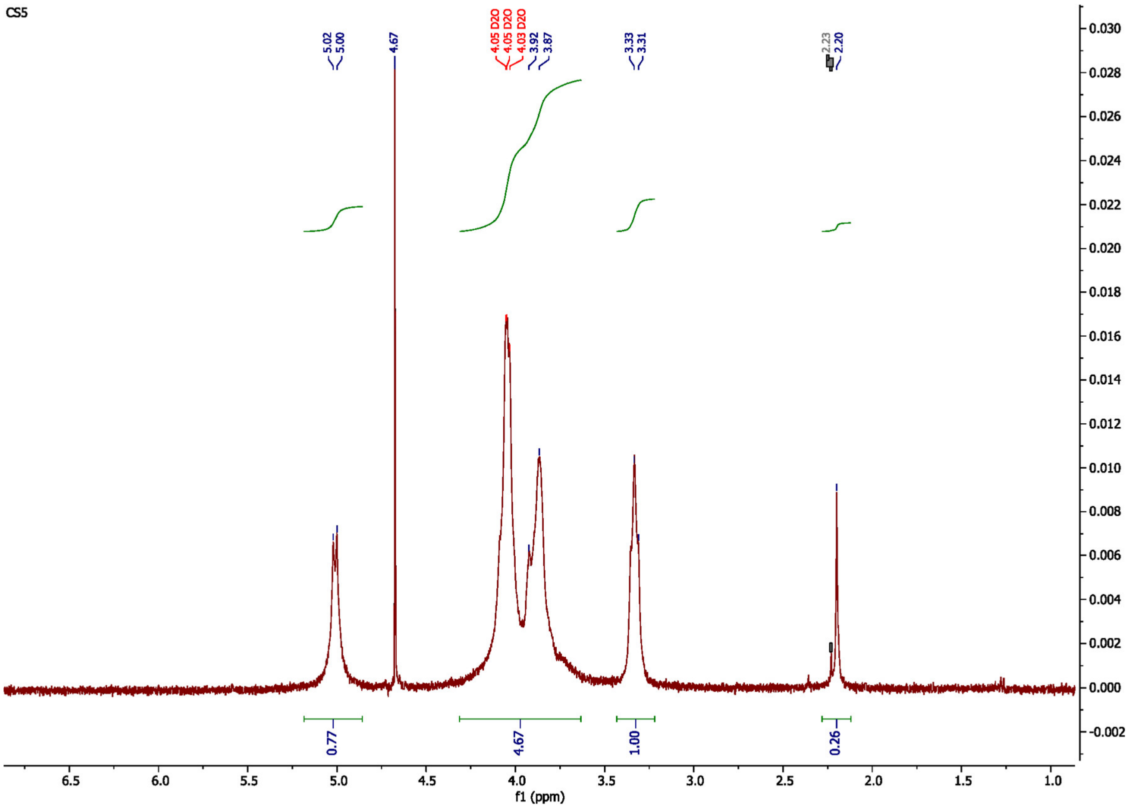
5.02
5.00
4.67
4.05 D2O
4.05 D2O
4.03 D2O
3.92
3.87
3.33
3.31
2.23
2.20
0.77
4.67
1.00
0.26
f1 (ppm)

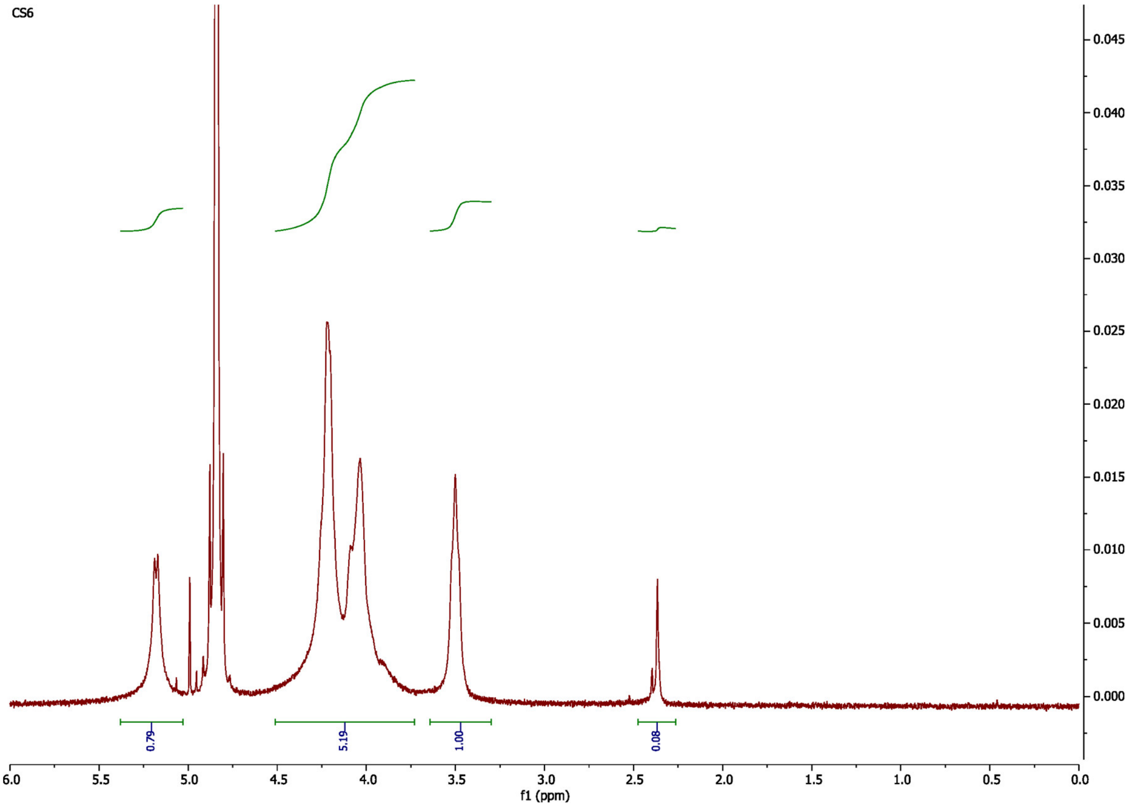

Table S1. Degree of deacetylation of chitosan's calculated from 1H- NMR spectra.

Sample name	DD (%)
CS1	82.33
CS2	80.33
CS3	97.3
CS4	96
CS5	91.33
CS6	97.3
